# Dietary Genistein Enhances Cognitive Performance in a Rabbit Model of Menopause

**DOI:** 10.1002/alz70855_106152

**Published:** 2025-12-24

**Authors:** Ezekiel A Irewole, Desheng Wang, Neha Lal, Logan D Bays, MacKinzie Smith, Delanie Talkington, Bernard G Schreurs

**Affiliations:** ^1^ West Virginia University, Morgantown, WV, USA

## Abstract

**Background:**

Systemic estrogen loss (menopause) is currently under investigation as a contributing factor in the observed increase in women's risk of Alzheimer's disease (AD). Despite the proven cognitive benefits and neuroprotective properties of endogenous estrogen, attempts to reduce AD risk by exogenous estrogen therapy have yielded controversial results. In this study, we investigated whether genistein, a phytoestrogen, can replace estrogen, rescue the effects of systemic estrogen loss on cognition, and potentially reduce AD risk.

**Method:**

We induced menopause via gonadectomy (GDX) in mature female rabbits and administered genistein through a special diet. Rabbits were assigned to one of three experimental groups: GDX with genistein diet (GDX+GN), GDX with control diet (GDX+CON), and sham GDX with control diet (sGDX+CON). All rabbits received their respective diets for eight weeks before undergoing cognitive assessment with trace eyeblink conditioning, a test of associative learning and memory.

**Result:**

The results show that dietary genistein improved learning and memory in gonadectomized rabbits across all cognitive indices, including the percentage of conditioned responses, onset latency, delayed memory recall, and response amplitude.

**Conclusion:**

Genistein may provide cognitive benefits in estrogen‐deficient conditions and may be considered in place of estrogen therapy to rescue cognitive impairment and reduce AD risk in post‐menopausal women.

